# The Pied Piper: A Parasitic Beetle’s Melodies Modulate Ant Behaviours

**DOI:** 10.1371/journal.pone.0130541

**Published:** 2015-07-08

**Authors:** Andrea Di Giulio, Emanuela Maurizi, Francesca Barbero, Marco Sala, Simone Fattorini, Emilio Balletto, Simona Bonelli

**Affiliations:** 1 Department of Science, University Roma Tre, Viale G. Marconi 446, I-00146 Rome, Italy; 2 Department of Life Sciences and Systems Biology, University of Turin, Via Accademia Albertina 13, I-10123 Turin, Italy; 3 CE3C—Centre for Ecology, Evolution and Environmental Changes / Azorean Biodiversity Group and Universidade dos Açores—Departamento de Ciências Agrárias, 9700-042 Angra do Heroísmo, Açores, Portugal; Universidade de São Paulo, Faculdade de Filosofia Ciências e Letras de Ribeirão Preto, BRAZIL

## Abstract

Ants use various communication channels to regulate their social organisation. The main channel that drives almost all the ants’ activities and behaviours is the chemical one, but it is long acknowledged that the acoustic channel also plays an important role. However, very little is known regarding exploitation of the acoustical channel by myrmecophile parasites to infiltrate the ant society. Among social parasites, the ant nest beetles (*Paussus*) are obligate myrmecophiles able to move throughout the colony at will and prey on the ants, surprisingly never eliciting aggression from the colonies. It has been recently postulated that stridulatory organs in *Paussus* might be evolved as an acoustic mechanism to interact with ants. Here, we survey the role of acoustic signals employed in the *Paussus* beetle-*Pheidole* ant system. Ants parasitised by *Paussus* beetles produce caste-specific stridulations. We found that *Paussus* can “speak” three different “languages”, each similar to sounds produced by different ant castes (workers, soldiers, queen). Playback experiments were used to test how host ants respond to the sounds emitted by *Paussus*. Our data suggest that, by mimicking the stridulations of the queen, *Paussus* is able to dupe the workers of its host and to be treated as royalty. This is the first report of acoustic mimicry in a beetle parasite of ants.

## Introduction

*“…and ere three shrill notes the pipe uttered, you heard as if an army muttered…*

*…from street to street he piped advancing, and step for step they followed dancing…”*

*Pied Piper of Hamelin*, Robert Browning (1842)

The ant colony is a heavily guarded, nearly impenetrable fortress rich with bountiful resources. Intruders attempting to infiltrate the ant society are immediately discovered via chemical cues, overtaken and dismantled. Nothing gets by, except for the few highly specialized, that have evolved the necessary chemical, morphological and behavioural tools to hack the complex recognition and communication system of the ants. Flying under the ant radar represents a huge boon that not only grants free access to the bounty of the colony—including the ants themselves—but further provides a safe and well-protected harbor to develop and live.

Among social insects, ants in particular are known to produce a variety of distinctive signals and use various communication channels to regulate their social organization [1]. The use of highly sophisticated communication systems is the key attribute that enables ants to act as a superorganism, thereby facilitating their dominance of terrestrial ecosystems [1].

The main channel of communication that drives almost all ant activities and behaviours is chemical, implemented through the use of semiochemicals (e.g. pheromones and cuticular hydrocarbons) [2]. It is thus not surprising that most invertebrate myrmecophiles have converged upon this channel to facilitate some level of protection or integration within the ant society. Indeed, chemically based protection for life with ants has independently evolved in a broad range of myrmecophilous invertebrates (e.g., pselaphine rove beetles [3] ant nest beetles [4,5], lycaenid butterflies [6], and spiders [7]).

**Fig 1:**
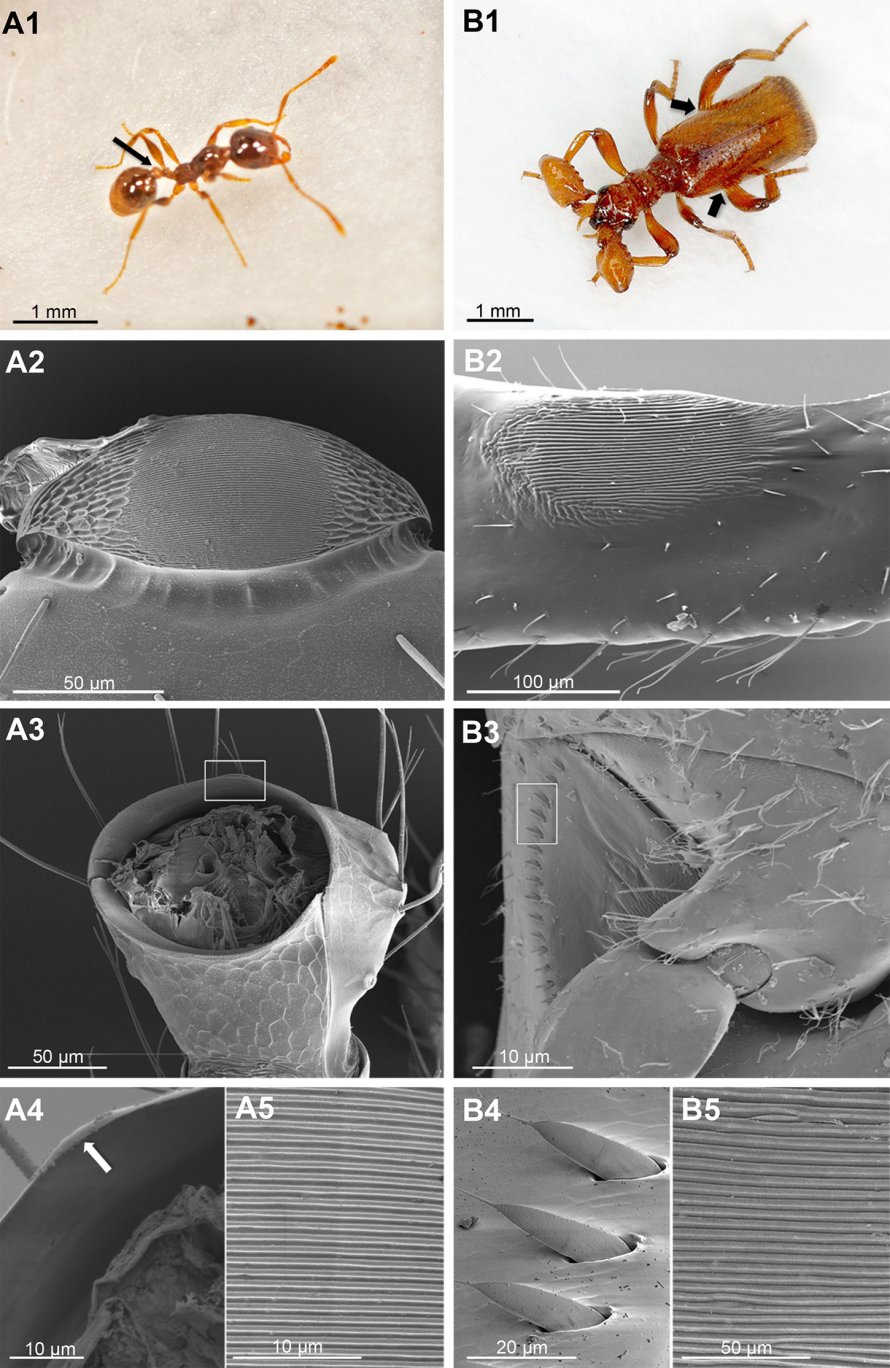
Comparative morphology of stridulatory organs of the ant *Pheidole pallidula* (a) and the beetle *Paussus favieri* (b). This figure shows clear similarities between the two structures. A1: *P*. *pallidula* worker (photo M. Muzzi), arrow indicates the position of the stridulatory organ. A2–A5: SEM micrographs of stridulatory organ of *P*. *pallidula* composed by a suboval and minutely ridged “file” (A2), placed on the meso-dorsal part of the first gastral segment (i.e. the fourth abdominal segment), and a “scraper” (A3, rectangular window), consisting of a medial cuticular prominence (A4, arrow) that originates from the posterior edge of the postpetiole (i.e. the third abdominal tergite) (A3); A5 represents a close-up of the ridged stridulatory file of A2. *P*. *pallidula* stridulates by quickly moving the gaster up and down. B1: *P*. *favieri* (male specimen, photo M. Muzzi), arrows indicate the position of the two stridulatory organs. B2–B5: SEM micrographs of right stridulatory organ of *P*. *favieri*, composed by a suboval, slightly raised and minutely ridged “file” (B2) positioned basally on the inner surface of the hind femora, rubbing against a “scraper” (B3) composed of a curved row of small cuticular spines (B4, close-up of rectangular window in B3) positioned at both sides of the proximal abdominal segment; B5 represents a close-up of the ridged stridulatory file of B2. *P*. *favieri* stridulates by shaking its posterior legs, singly or in combination.

However, it has been long acknowledged that the acoustic channel of communication also plays an important role in the organization of ant societies (see references in [8 **–** 10]). Adults of Ponerinae, Nothomyrmecinae, Pseudomyrmecinae and Myrmecinae ants are able to produce stridulations consisting of low frequency sounds (a series of repeated ‘chirps’), by rubbing a tergal carena, called the “scraper” or *plectrum* (Fig 1, 1A3 and 1A4), against a minutely ridged area, called the “file” or *pars stridens* (Fig 1, 1A2 and 1A5), placed between two abdominal segments [1,11,12]. Ants produce such acoustical signals for various purposes, including social organization, recruitment, mating, or help request [13].

Very little is known regarding exploitation of the acoustical channel by myrmecophiles. What is clear, however, is that acoustical mimicry raises the hierarchical status of a social parasite once it is successfully integrated into the society via chemical mimicry [8,13]. Among the thousands of known invertebrate myrmecophiles that have exploited the chemical communication of ants (see [14]), to date only a single instance of acoustic exploitation is known [8,13].

Immature stages of the lycaenid butterfly, *Maculinea*, are social parasites of ants, living within the brood chambers of the ant colony where they solicit and receive care from workers.

It was recently demonstrated that the key to *Maculinea* attention and social status within the colony was not their semiochemicals that mimic the cuticular hydrocarbons of their hosts, rather it was their ability to produce stridulations that mimic those of the queen ant thereby interfering with their host’s acoustical communication system. To date, *Maculinea* is the only known insect that uses also the acoustic channel to parasitize ants.

Among social parasites of ants, the ant nest beetles (Coleoptera, Carabidae, Paussini) stand out as arguably the most ecologically fascinating and phenotypically bizarre [15]. Ant nest beetles are obligate myrmecophiles during all life stages and are fully integrated into their host ant society through means of hacking the hosts’ chemical communication [4,5,16,17]. The ant nest beetles of the genus *Paussus* are known to deliver appeasement chemicals stored in trichomes adorning their body [18] to their host ants and in return receive full access to the colony and its bounty. Once integrated, adult *Paussus* move throughout the colony at will and prey upon the adult ants and especially their brood, surprisingly never eliciting aggression from the ants [5]. This symbiosis has resulted in one of the most stunning examples of rapid adaptive radiation known to date [15].

Unlike *Maculinea* butterflies, *Paussus* depends on ants during all stages of its development, thus adaptations for social parasitism must be innervated in both adults and their virtually immobile immatures [19]. Furthermore, *Paussus* adults interact directly with the queen in intimate ways previously unknown in myrmecophiles [5].

That *Paussus* are able to prey upon brood and adults alike and interact with the queen without eliciting any aggressive behavior from the ants (including those being preyed upon!) [4,5] demonstrates the shear sophistication of their masquerade parasitism and suggests that *Paussus* have utilized another channel beyond chemical mimicry to achieve their hierarchical standing among their hosts.

*Paussus* beetles are rare and difficult to rear, and much of their anatomy and behaviours remains unclear [4].

The presence of stridulatory organs in *Paussus* has long been known [20–23] but only recently described in detail and interpreted as a mechanism possibly evolved to interact with ants [24].

We tested this hypothesis by conducting a thorough investigation of the stridulation and behaviour of *Paussus favieri* and its host ant, *Pheidole pallidula*. We first characterized the acoustic signals of *Paussus favieri* and worker, soldier and queen castes of its host ant, *Pheidole pallidula*. We next analysed ants’ responses to *Paussus* stridulations by playback experiments and found that the ants react to *Paussus* sounds, which demonstrates that *Paussus* stridulations convey an interspecific message. More unexpected, however, was the finding that *Paussus* beetles were able to produce at least three different acoustical signals that elicited different responses in the ants. We show that *Paussus favieri* is not only able to mimic ants’ stridulations but can also “speak” three different “languages”, each corresponding to sounds produced by different ant castes, including the queen, to direct host ant behaviours. In effect, *Paussus* utilizes modular acoustical mimicry to achieve the highest hierarchical status of the colony.

In presenting this new finding regarding adult beetles we document that *Paussus* has evolved the most sophisticated acoustical behaviour for social parasitism known to date.

## Materials and Methods

### Ethics Statement

*Paussus* beetles and *Pheidole pallidula* ants were collected in Morocco under permit from the Institut Scientifique, Université Mohammed V Agdal (Rabat, Morocco), released from the Director Prof. A. El Hassani in 2010. The species are not protected nor endangered. They were kept in the lab in artificial nests under sub-natural, controlled environmental conditions and treated according to the highest ethical standards in order to limit the animal stress to the minimum.

### Insect collection and laboratory breeding

*Paussus favieri* specimens were collected inside the nest of *Pheidole pallidula* under stones in a Mediterranean garigue on the High Atlas Mountains (Tizi-n-Test, about 3 km N to pass, 2063 m elevation, 30.87288° N– 8.36204° W), in May 2010. Adults of *Paussus favieri* were reared with ants from the nest in which they were found; when multiple specimens of *Paussus favieri* were found in the same nest, all specimens were reared together. The colonies were kept in climate controlled conditions with a day:night cycle of 12:12 h, a temperature cycle of 21–24°C and a moisture about 60–70%. Five colonies were housed in artificial nests consisting in transparent glass boxes (32×22×15 cm) lined with a layer of plaster (18 × 22 × 4 cm thick), and the walls were coated with Fluon (BioQuip) to prevent ants from escaping. The plaster floor was separated in some chambers, and a hole gave access to the foraging area. The plaster was regularly moistened, and the ants were fed with diluted sugar or honey, fruit flies or moth caterpillars provided three times per week and water *ad libitum*. After the ants and beetles were acclimated to these new conditions (for about 10 days), behavioural observations were made.

### Morphological analyses

The stridulatory organs of beetles and ants were prepared as described in Di Giulio et al. [16] and studied by a FIB/SEM Helios NanoLab (FEI Company) equipment in the LIME (Interdepartmental Elecron Microscopy Lab) lab of Roma Tre University (Rome, Italy). The material is preserved in the A. Di Giulio collection (Rome, Italy).

### Sound recording

Stridulations produced by *Paussus favieri* (5 males and 4 females) and *Pheidole pallidula* (5 workers, also known as ‘minor workers’; 5 soldiers, also known as ‘major workers’; and 5 queens, from different colonies) were recorded. For each stridulations, we recorded the dominant frequency (Hz), pulse length (s) and sound intensity (dB). In total, we analysed 276 sound sequence measurements: queens (66), workers (103) and soldiers (107). 482 pulses were examined for *Paussus favieri*. Samples were recorded for 10 minute periods, starting 5 minutes after an individual was introduced in the recording chambers and had become calm. During recording sessions, the specimens were placed on the microphone. Beetles and ants were recorded by using a custom recording equipment consisting of a 12.5 × 8 × 2 cm recording chamber with a moving-coil miniature microphone attached through the centre (sampling rate set to 44.10 kHz). A second microphone was used to record in anti-phase the ambient noise. The microphone output signal was processed through a two-stage low-noise amplification using a SP-24 B stereo microphone preamplifier (gain 53 dB). Segments containing acoustic recordings were digitally saved in WAV format (16-bit amplitude resolution) on a laptop computer using Audacity v. 1.2.4 (http://audacity.sourceforge.net/). The equipment was powered by a 12V gel cell battery, and the recording chamber and microphones were located inside an anechoic chamber to further reduce ambient noise and interference [8]. Residual background noise was removed using iZotope Rx software v1.20.590. For each file, the waveform and FFT spectrogram (FFT size = 512; Hanning window shape) were generated using Audacity v. 1.2.4. Values of recorded parameters are given in Supplementary Information (S1 Dataset).

### Statistical analysis of sound characteristics

In *Paussus* stridulations, we identified three types of pulses and analysed separately the acoustic characteristics of each type of pulse. Based on the three sound parameters, single pulses were ordered by principal components analysis (PCA) using R 3.1.2 (http://www.R-project.org/).

To test whether sound differed between groups, we calculated the pairwise Euclidean distance over all standardised parameters and used a nested (‘individuals’ within ‘groups’) ANOSIM implemented in Primer v6 (Primer-E Ltd.). The sound parameters were log (*x*+1) transformed. We calculated the average pairwise distances and used a two-sample t-test to compare differences between group distances. Data were analysed using a SPSS package ver. 20.0. We included in similarity analyses (see above) also sound parameters of stridulations emitted by *Myrmica scabrinodis* queens and workers as control groups. Samples of *Myrmica* ants, collected at Caselette (Turin, Italy, 45.12778° N –7.49056° E), have been recorded using the aforementioned recording device with the same experimental settings.

### Playback experiments

Playback assays were carried out in seven artificial arenas, with a 7 cm diameter base. Speakers were attached in the central zone of the base through a hole, sealed on the outside with Blu-tac and, covered with a thin layer of sand to resemble the natural condition.

Two workers, from the same *Pheidole pallidula* colony, were placed in each arena and allowed to settle for 10 minutes before being played one of the following seven sound tests: (1) *Paussus favieri* strings of pulses (composed of Pa and Pb); (2) *Paussus favieri* single pulses (Pc); (3) *Pheidole pallidula* queen; (4) *Pheidole pallidula* soldiers; (5) *Pheidole pallidula* workers; and (6) two controls (white noise and silent speaker). The sounds were produced by MP3 players playing loops of the original recordings, with each volume adjusted to the natural level by attaching the speaker to the microphone of the recording equipment and by calibrating to the same levels reached during the recording.

We used simultaneously seven arenas (boxes), each with two worker ants subject to one of the seven different sound stimuli mentioned above (Pa+Pb, Pc, queen, soldiers, workers, white noise and silence).

We observed in succession each box for 30 s; i.e., we observed the first box for 30 s, then we observed the second box for 30 s, and so on. After all the seven boxes have been observed for 30 s each, we started a new set of observations with the same procedure; in total, we performed 6 sets of observations. Thus, each box was observed for 180 s and the experiment section lasted 21 minutes (180 s for 7 boxes). During each 30 s observation, we recorded the presence of the following behaviours: (1) *walking* (the worker was attracted to the speaker and walked over it); (2) *antennating* (the worker antennated the speaker for at least 3 s); (3) *guarding* (the worker showed an alert on-guard poise (*sensu* [8]) on the speaker for at least 3 s; (4) *digging* (the worker dug into the soil over the speaker); (5) *staying* (workers stayed on the speaker without performing any movement or assuming a particular poise). The source of sound for each box was randomly assigned before each of the 21 minute sections and, to avoid any prejudice, the observer was not informed about which stimulus was assigned to each box. After each of these 21 minute sections, new sand was introduced and all equipment, including speakers and arenas, was cleaned with absolute alcohol and rinsed with distilled water. Each of the 10 colonies was tested twice, which led to a total of 420 minutes of observations. Playback recording data are given in Supporting Information (S1 Dataset).

To test differences in counts of worker behavioural responses to different sounds, we fitted a Generalised Linear Models (SPSS v. 20.0 software) with a factor reflecting the sound sources, and a second factor expressing the identity of trailed colonies to control for the typical overall differences in activity among individual colonies (normal errors, identity link). Pairwise differences among groups were established using Tukey HSD (Honestly Significant Difference) tests and average frequency values were plotted (S1 Table). Recordings and playback bioassays have been performed using the facilities of the University of Torino.

## Results and Discussion

### Diversity and overlap of *Paussus* and host ant stridulations

**Fig 2:**
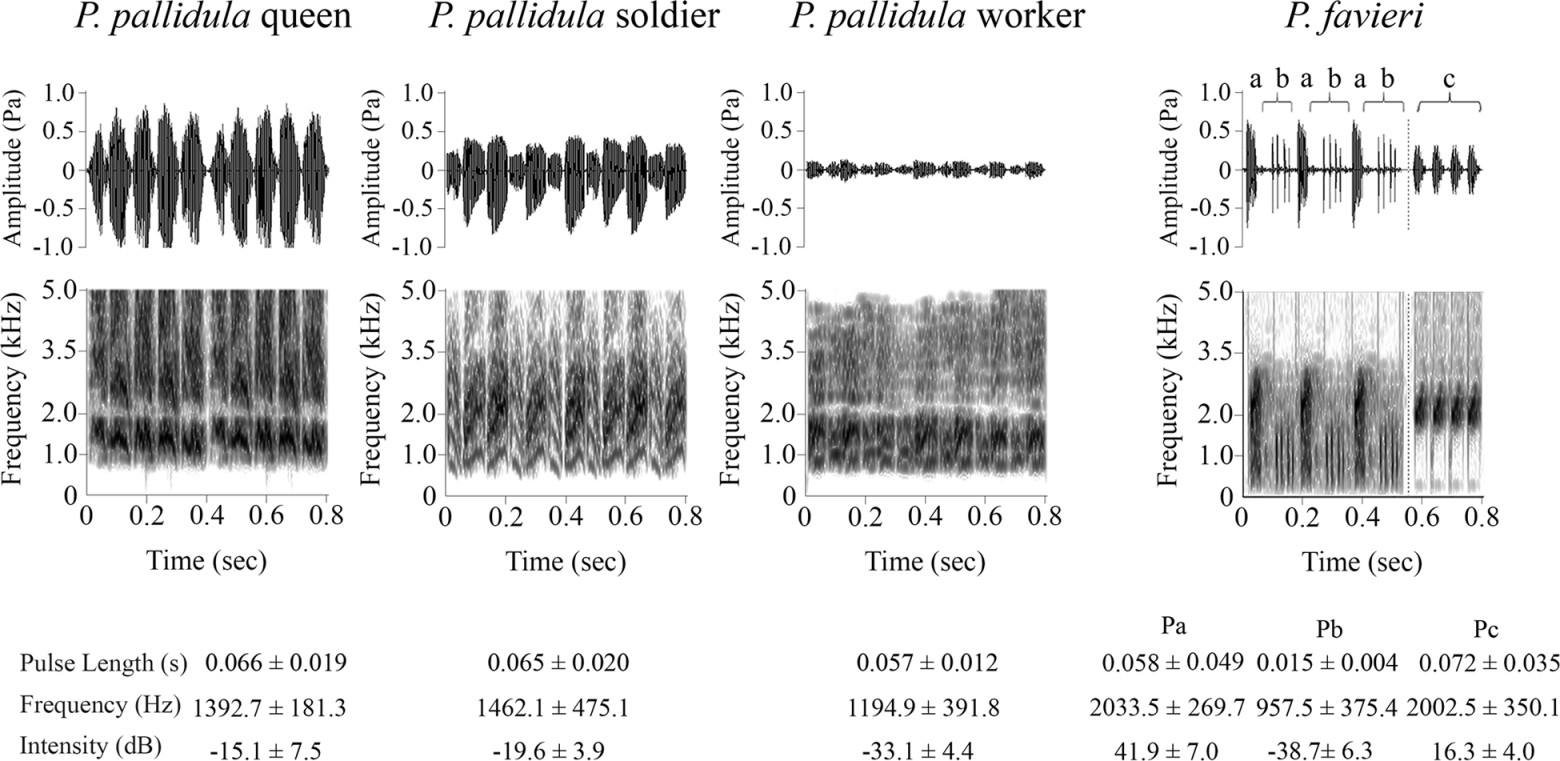
Waveforms and spectrograms of stridulations emitted by *Pheidole pallidula* castes and *Paussus favieri*. Waveforms of stridulations emitted by three *Pheidole pallidula* castes (queen, soldier and worker) and by *Paussus favieri* (Pulses a-b-c) are shown in the upper part of the figure. Pulses a (Pa), which are more similar to the sounds emitted by soldiers and workers, and Pulses b (Pb), which are similar to the sounds emitted by workers, are found alternating in trains. Pulses c (Pc) are closer to queens’ stridulations and are emitted subsequently, to form different trains. The lower part of the figure shows the corresponding spectrograms: darker parts correspond to higher energy densities while lighter parts correspond to lower energy densities.

We found that members of all three castes (queen, workers, and soldiers) of *Pheidole pallidula* produce with their abdominal stridulatory organs (Fig 1, 1A1–1A5) distinctive sounds (Fig 2 and S1–S3 Files). Stridulations consist of a series of pulses repeated from a few to about ten seconds, without any interruption. Trains of pulses of both queens and soldiers contain about twenty pulses, while the workers’ trains are generally longer with about thirty pulses.

Whereas queen and soldier sounds were not distinguishable on the basis of pulse length and frequency, all ant castes differed in sound intensity. An additional table file shows this in more detail (S2 Table).

All individuals of both sexes of *Paussus favieri* are able to emit stridulations (Fig 2 and S4 File). These stridulations are produced by a slightly different mechanism from that of ants. The ridges of the beetle’s file are located on the hind femora (Fig 1, 1B2 and 1B5) and are about five times farther apart than those of the ant, which are located on the first gastral (fourth abdominal) segment (Fig 1, 1A2 and 1A5). Moreover, the beetle uses a row of scrapers (placed on the proximal abdominal segment) (Fig 1, 1B3 and 1B4) rather than the single scraper (placed on the postpetiole, i.e. the third abdominal tergite) used by the ant (Fig 1, 1A3 and 1A4). These stridulations consist of three different kinds of pulses (Fig 2), which we call Pa, Pb and Pc. Pa and Pb alternate in trains, while Pc pulses are emitted subsequently, forming a separate sequence. Sequences of pulses lasted approximately 3 seconds, on average. All studied beetles of both sexes emitted all three types of signals. The use of a Mann-Whitney test to compare pulse parameters (i.e., Pulse Length, Frequency and Intensity), as well as the various types of pulses (Pa, Pb and P), revealed no differences between female and male acoustics (*P*>0.05 in all cases).

To account for individual differences in the sound emission, we analyzed variations in each sound component (Pulse length, Dominant Frequency and Intensity) by using Generalised Linear Models (GLM) in which beetle’s pulses (Pa, Pb, and Pc) were used as fixed factor and the “individual” was selected as random factor. We found no effect of individual identity (GLM results: F_482, 8_ = 1.561, *P* = 0.0.183 for Pulse Length; F_482, 8_ = 1.115, *P* = 0.400 for Frequency; F_482, 8_ = 0.678, *P* = 0.705 for Intensity), whereas each sound component differed among *Paussus* pulses (F_482, 2_ = 967.725, *P* < 0.001 for Pulse Length; F_482, 2_ = 581.164, *P* < 0.001 for Frequency; F_482, 2_ = 1937.577, *P* < 0.001 for Intensity). Univariate analysis of each sound component showed that the three types of pulses (Pa, Pb, and Pc) emitted by *Paussus favieri* were significantly different, except for the dominant frequency measured on Pa and Pc (S2 Table). Also *Pheidole pallidula* ants produced stridulations that did not differ among individuals (ANOVA: *P* > 0.05) but are cast specific (ANOVA: F_296,2_ = 9.007, *P* <0.001 for Pulse length; F_296,2_ = 13.447, *P* < 0.001 for Frequency; F_296,2_ = 296.694, *P* <0.001 for Intensity).

A comparison of *Paussus favieri* and *Pheidole pallidula* sounds revealed that pulse Pa produced by the beetle had the same pulse length as those emitted by soldiers and workers, while pulse Pc emitted by the beetle had the same intensity and pulse length as that of the queens (S2 Table).

**Fig 3:**
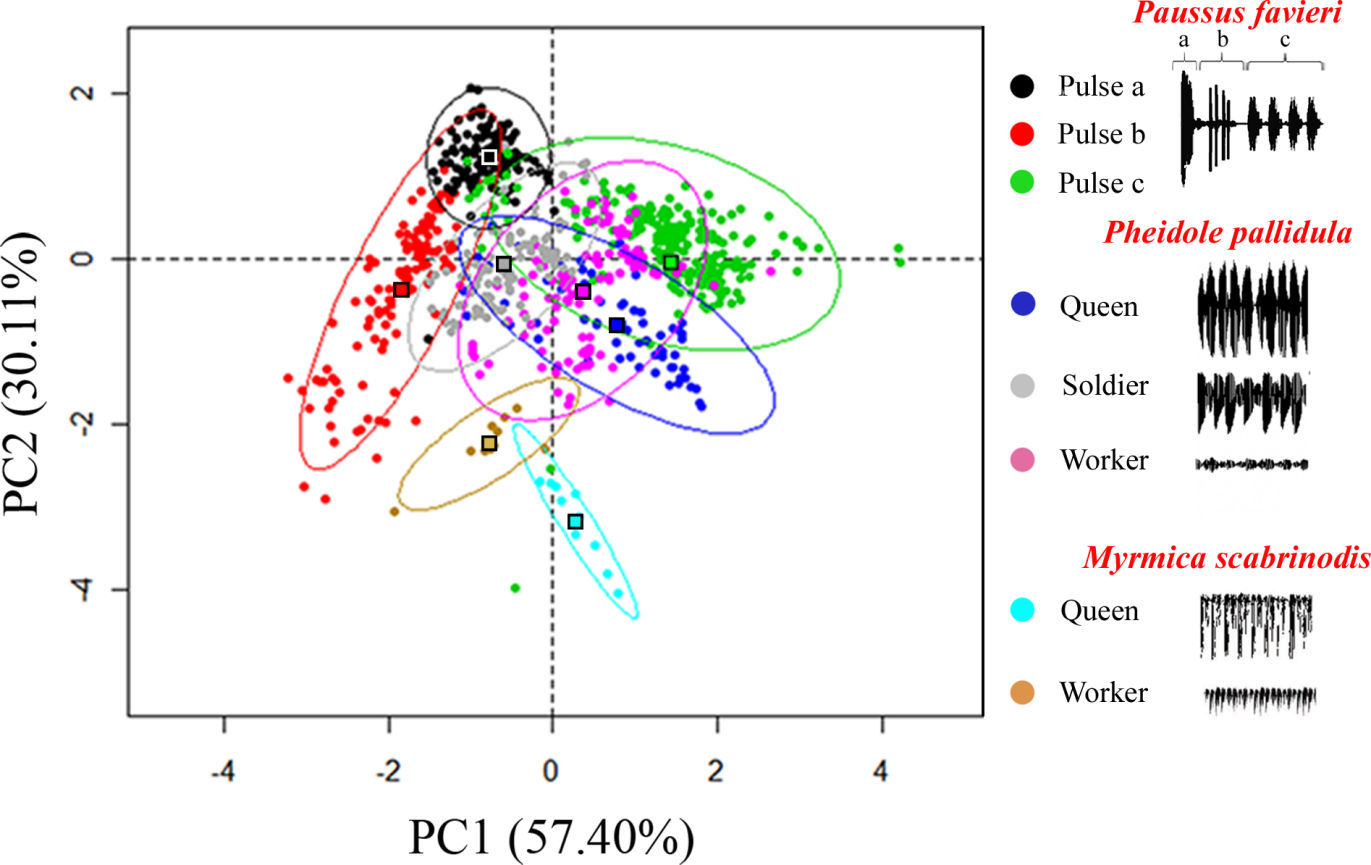
Principal Components Analysis (PCA) of acoustic parameters. Combined effects of the three sound parameters (pulse length, frequency, and intensity) shown as the first and second component plot of a principal components analysis over all individual pulse measurements; ovals indicate 95% confidence intervals; filled squares indicate the centroid for each group. Sounds produced by *Myrmica* queen and worker ants are also plotted for comparison. The first component explained 57% of total variance and was mainly influenced by pulse length (waveform parameter). The second component accounted for 30% of total variance and significantly discriminated groups on the basis of peak frequency (spectral parameter). Component loadings of PC1: Pulse length: 0.893, Frequency: 0.602; Intensity: -0.749. Component loadings of PC2: Pulse length: -0.042, Frequency: 0.764; Intensity: 0.564. Univariate analysis of variance (ANOVA) performed on PCA factors showed significant difference among groups (see text).

The three sound parameters were also subject to a Principal Components Analysis (PCA) with stridulations of the ant *Myrmica scabrinodis* used as a control. A PCA of all individual measurements showed that sounds produced by *Pheidole pallidula* and *Paussus favieri* can be divided into 6 separate acoustic groups corresponding to the three ant castes, with only slight overlap between queens and soldiers, and to the three kinds of *P*. *favieri* pulses, with a deep separation of Pc from Pa and Pb (Fig 3; results of ANOVA performed on the first two components extracted by the PCA: F _778,7_ = 354.561, *P* < 0.001 for the first component; and F _778,7_ = 179.204, *P* < 0.001 for the second component). As expected, stridulations emitted by *Myrmica* ants grouped apart from both *Pheidole pallidula* and *Paussus favieri*, suggesting the ability of the beetle to imitate its host ant stridulation more than other ants’ acoustical emissions.

A nested analysis of similarity (ANOSIM) of the Euclidean distance matrix demonstrated a separation between signals produced by queens, soldiers, workers, *Paussus favieri* Pa, Pb, Pc and *Myrmica* queens and workers (overall: R = 0.994, *P* < 0.001). Patterns of similarity in the standardised Euclidean distances of stridulations revealed important interspecific similarities between *Paussus* and their host ants (Tables 1–2). In particular, Pa was equally similar to stridulations by *P*. *pallidula* soldiers and workers, and less similar to Pc and Pb (Table 2). Pb was closer to the stridulations of workers’ than soldiers' or to Pa stridulations emitted by *P*. *favieri* itself (Table 2). Pc stridulations were significantly more similar to *P*. *pallidula* queens than to soldier and to Pa stridulations (Table 2). *Paussus* and *Pheidole* stridulations were always very different from those emitted by *Myrmica* queens and workers (Table 2). Thus we can conclude that each of the three types of pulse emitted by *P*. *favieri* is always more similar to a stridulation emitted by a specific ant caste than to any other type of *Paussus favieri* pulses. *Paussus* trains of Pa and Pb are similar to those of either ant soldiers or workers (the shortest distance was found between Pb and *Pheidole pallidula* workers), while single pulses (Pc) are more similar to stridulations emitted by queens.

**Table 1:** Euclidean distances between sounds emitted by parasite and ants.

	Pb*	Pc*	Q^&^	S^&^	W^&^	Qm^#^	Wm^#^
**Pa** *	0.365 ± 0.042	0.353 ± 0.038	0.495 ± 0.028	0.299 ± 0.051	0.305 ± 0.047	0.996 ± 0.055	0.699 ± 0.017
**Pb** *	─	0.485 ± 0.053	0.478 ± 0.035	0.354 ± 0.033	0.134 ± 0.043	0.851 ± 0.055	0.555 ± 0.082
**Pc** *	─	─	0.170 ± 0.035	0.207 ± 0.029	0.356 ± 0.043	0.710 ± 0.055	0.500 ± 0.016
**Q** ^**&**^	─	─	─	0.148 ± 0.027	0.377 ± 0.031	0.515 ± 0.063	0.661 ± 0.265
**S** ^**&**^	─	─	─	─	0.240 ± 0.019	0.637 ± 0.061	0.603 ± 0.228
**W** ^**&**^	─	─	─	─	─	0.809 ± 0.058	0.594 ± 0.139
**Qm** ^**#**^	─	─	─	─	─	─	0.357 ± 0.064

Euclidean distances (mean ± SD) were calculated using the three standardised sounds parameters, peak frequency (Hz), intensity (dB) and pulse length (s), produced by by *P*. *favieri*, its host ant *Pheidole pallidula* and ant control group.

*Paussus favieri: Pa = pulse a; Pb = pulse b and Pc = pulse c.

^*&*^ *Pheidole pallidula*: Q = queens; W = workers and S = soldiers.

^*#*^ *Myrmica scabrinodis*: Qm = queens and Wm = workers.

**Table 2:** Pairwise comparisons computed on mean Euclidean distances of sounds emitted by ants and parasite.

**Q**	**Q-Pc**	**Q-W**	**Q-Pb**	**Q-Pa**	**Q-Qm**	**Q-Wm**
**Q-S**	1.936	18.984**	28.967**	32.557**	24.718**	5.875**
**Q-Pc**	─	19.860**	31.957**	37.126**	25.200**	9.781**
**Q-W**	─	─	9.679**	12.237**	11.016**	4.246**
**Q-Pb**	─	─	─	1.905	4.668**	3.643**
**Q-Pa**	─	─	─	─	3.694**	3.362**
**Q-Qm**	─	─	─	─	─	1.035
**S**	**S-Pc**	**S-W**	**S-Pa**	**S-Pb**	**S-Wm**	**S-Qm**
**S-Q**	5.535**	8.907**	8.519**	18.449**	6.040**	21.432**
**S-Pc**	─	4.457**	8.237**	17.249**	9.203**	26.456**
**S-W**	─	─	4.355**	12.094**	6.361**	23.391**
**S-Pa**	─	─	─	4.640**	6.545**	12.681**
**S-Pb**	─	─	─	─	5.715**	16.048**
**S-Wm**	─	─	─	─	─	0.358
**W**	**W-S**	**W-Pa**	**W-Pc**	**W-Q**	**W-Wm**	**W-Qm**
**W-Pb**	9.137**	17.998**	24.352**	20.048**	18.945**	41.905**
**W-S**	─	5.232**	10.052**	14.716**	9.856**	35.728**
**W-Pa**	─	─	5.314**	5.524**	11.582**	25.794**
**W-Pc**	─	─	─	2.088*	9.844**	28.317**
**W-Q**	─	─	─	─	5.913**	24.472**
**W-Wm**	─	─	─	─	─	4.531**
**Pa**	**Pa-W**	**Pa-Pc**	**Pa-Pb**	**Pa-Q**	**Pa-Wm**	**Pa-Qm**
**Pa-S**	0.492	5.758**	6.576**	17.417**	32.027**	42.682**
**Pa-W**	─	5.812**	7.044**	18.967**	34.528**	50.141**
**Pa-Pc**	─	─	1.861	20.428**	37.382**	59.093**
**Pa-Pb**	─	─	─	14.852**	32.739**	53.956**
**Pa-Q**	─	─	─	─	27.094**	39.862**
**Pa-Wm**	─	─	─	─	─	21.740**
**Pb**	**Pb-S**	**Pb-Pa**	**Pb-Q**	**Pb-Pc**	**Pb-Wm**	**Pb-Qm**
**Pb-W**	22.594	28.849**	36.590**	40.206**	26.585**	54.889**
**Pb-S**	─	1.360	13.239**	12.106**	11.454**	37.822**
**Pb-Pa**	─	─	12.515**	16.011**	14.157**	41.688**
**Pb-Q**	─	─	─	0.652	4.311**	27.756**
**Pb-Pc**	─	─	─	─	4.516**	25.758**
**Pb-Wm**	─	─	─	─	─	12.791**
**Pc**	**Pc-S**	**Pc-Pa**	**Pc-W**	**Pc-Pb**	**Pc-Wm**	**Pc-Qm**
**Pc-Q**	4.222**	22.800**	19.879**	28.874**	22.532**	40.014**
**Pc-S**	─	20.809**	15.921**	26.022**	21.188**	39.878**
**Pc-Pa**	─	─	0.371	18.248**	14.785**	32.850**
**Pc-W**	─	─	─	14.890**	9.678**	27.183**
**Pc-Pb**	─	─	─	─	2.467*	15.730**
**Pc-Wm**	─	─	─	─	─	8.562**

Differences computed by two-sample t-tests in the average pairwise Euclidean distances, reported in Table 1, between groups of sounds produced by *P*. *favieri*, its host ant *Pheidole pallidula* and *Myrmica scabrinodis*. T-test values are differently ordered in each table to show in the diagonals series of increasing distances between a type of sound (e.g. **Pa**) and all the other sound stimuli (Pb, Pc, Q, S, W, Qm, Wm): **Pa-**S = **Pa-**W < **Pa-**Pc = **Pa-**Pb < **Pa-**Q < **Pa-**Wm < **Pa-**Qm; **Pb-**W < **Pb-**S = **Pb-**Pa < **Pb-**Q = **Pb-**Pc < **Pb-**Wm < **Pb-**Qm; **Pc-**Q < **Pc-**S < **Pc-**Pa = **Pc-**W < **Pc-**Pb < **Pc-**Wm < **Pc-**Qm; **Q-**S = **Q-**Pc < **Q-**W< **Q-**Pb = **Q-**Pa < **Q-**Qm = **Q-**Wm; **S-**Q < **S-**Pc < **S-**W < **S-**Pa< **S-**Pb < **S-**Wm = **S-**Qm; **W-**Pb < **W-**S < **W-**Pa < **W-**Pc < **W-**Q < **W-**Wm < **W-**Qm (< stands for statistical differences in the mean distances between the groups, while = means no differences).

*P<0.05

**P<0.01

### Function and modularity of *Paussus* stridulations

To assess the effects of *Paussus favieri* stridulations on ant behaviour, we performed a series of playback experiments, where the only stimuli presented to workers were the acoustic emissions produced by one of the following: (1) the beetle (single pulses—Pc—and trains—Pa and Pb—as separate stimuli) and by (2) queens, (3) soldiers and (4) workers in separate experimental trials. As controls we used both a silenced headphone and a computer generated white noise.

**Fig 4:**
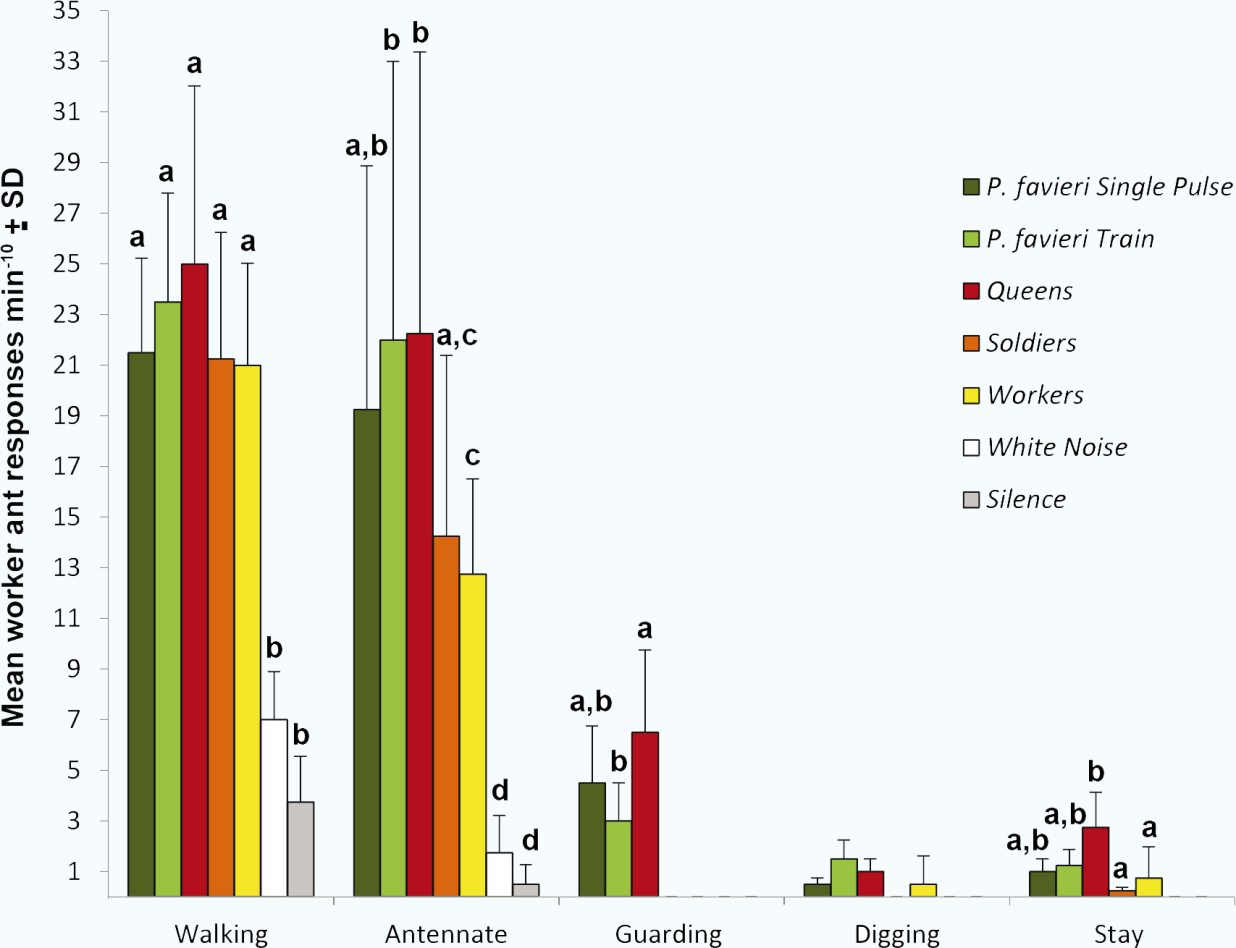
Playback experiments. Behavioural responses of *Pheidole pallidula* colonies to sound recordings of *Paussus favieri* (single pulses), *P*. *favieri* (trains), *Pheidole pallidula* queens, *P*. *pallidula* soldiers, *P*. *pallidula* workers, and to two controls (white noise and silence) are shown. Five benevolent behaviours were observed; no antagonistic behaviour was observed. GLM testing for the effect of sounds and colony showed a significant overall difference in responses occurred within five behaviours (N = 140; Walking: F_stimuli_ = 22.623, df = 6, *P* < 0.001, F_nest_ = 1.253, df = 9, *P* = 0.284; Antennate: F _stimuli_ = 39.414, df = 6, *P* < 0.001, F_nest_ = 1.221, df = 9, *P* = 0.302; Guarding: F _stimuli_ = 12.942, df = 6, *P* < 0.001, F_nest_ = 1.388, df = 9, *P* = 0.216; Digging: F_stimuli_ = 2.667, df = 6, *P* = 0.024, F_nest_ = 0.667, df = 9, *P* = 0.735; Staying: F_stimuli_ = 5.083, df = 6, *P* = 0.004, F_nest_ = 1.856, df = 9, *P* = 0.079). The letters above each column indicate significance (*P* < 0.05) in pairwise *post hoc* Tukey tests. The same letter indicates no significant difference within each type of behaviour.

In playback experiments, we did not observe any antagonistic or alarmed ant behaviour, but always non-aggressive responses involving attraction (walking, antennating and staying) and interaction with other ant castes (guarding, digging). A GLM analysis showed that workers’ reactions to the sound stimuli were significantly different from controls for all observed behaviours. Behaviours such as guarding, digging and staying were not produced by controls (Fig 4). *Pheidole pallidula* ants were attracted to and induced to “walk” on the speaker by all the sound stimuli, showing no differences in the frequency of responses to the beetle’s or ant castes’ stridulations. Interestingly, playback of *P*. *favieri*’s stridulations produced a number of antennations similar to that elicited by sounds emitted by *Pheidole pallidula* queens. Soldiers’ stridulations elicited a smaller amount of antennations, but not statistically different from those elicited by *Paussus favieri* single pulses or worker stridulations. Results for antennation are remarkable because this behaviour is known to be linked to nest-mate recognition, recruitment, or to facilitate trophallaxis or pheromone emission [25 **–** 27]. Guarding was only induced by *Paussus favieri* and queens’ sounds. Workers responded to these stimuli by assuming a posture similar to that adopted when they attended queens or objects of great value to their society [2,28]. The queen’s sounds produced the highest occurrences of guarding, which is consistent with the high status and protection afforded to queens in the colony’s hierarchy. One of the most stunning results of the present study is that the emission of single pulses (Pc) of *Paussus favieri* elicited in worker ants a guarding behaviour that is statistically no different than when presented with emissions of the queen (Fig 4). These findings, together with the highest similarity between Pc and queen stridulations (demonstrated by both uni- and multivariate analyses), support our hypothesis that single pulses are used by the beetle to be treated like a queen by its host ants.

A rarely elicited behaviour was digging, which is commonly induced in nest-mates by ants trapped under the soil, for instance after the collapse of a tunnel [29,30]. *Atta* ants, for example, are known to emit rescue calls when trapped [31]. Here we observed that both the sounds of *P*. *favieri* and those of queens and workers induced a digging behaviour in *P*. *pallidula* workers. Trains of *P*. *favieri* pulses seem to elicit digging, at least more often than the worker’s stridulations did (Fig 4). Except for the two controls, all sound stimuli caused workers to stand on the speakers. Sounds produced by queen ants induced a similar amount of staying on the speaker as the two sounds (trains and single pulses) emitted by *Paussus favieri* and a statistically higher amount of staying responses than those elicited by the conspecific soldiers’ and workers’ calls. We hypothesise that staying on the speaker might be related to the ant’s perception of vibrations, which is likely to occur along the legs, as shown in carpenter ants [32].

Findings provided by the analysis of ant-beetle acoustical patterns coupled with ethological observations reveal that a particularly complex form of communication occurs between the beetle (*Paussus favieri*) and the host ant (*Pheidole pallidula*). This represents the second known case of acoustic mimicry in myrmecophilous organisms (the first one being that between *Maculinea* butterflies and *Myrmica* ants), and the first one for beetles.

Although these models involve phylogenetically very distant taxa, and different life stages (larvae and pupae in lycaenids and adults in *P*. *favieri*), several similarities are surprisingly evident. In both cases queen’s stridulations are different from those of the other castes, in particular from those of workers. This clear distinction among castes generates the opportunity for the parasite to selectively imitate each specific caste [8]. *Maculinea* immatures exploit this opportunity to an extent by emitting stridulations that are more similar to those of the queen than to those of workers and thereby gain higher rank within the colony [8]. Also *Myrmica’s* sclerotised pupae used sounds to signal their social status within the colony’s hierarchy [9]. Queen-like stridulations produced by *Maculinea* immatures are an effective strategy to cope with their need to be foraged, attended and saved in case of colony disturbance during their development inside the ant nest. The butterfly larvae mostly remain still and hidden in the brood chambers, asking for food and care, thus encompassing a basically invariant interaction during all the time (11–23 months) the parasite lives within the ant colonies.

In contrast, the strategy of *Paussus* is much more complex, because it involves multiple types of interaction. Adult beetles need to enter the nest, move freely within it to find appropriate places for egg laying and larval development, feed on the ant broods without being attacked, rewarding the ants by supplying attractive substances from their glandular trichomes, and find their partners for mating inside the nest. Thus, they need to interact with different castes in different ways, due to the allocation of the behavioural repertoire of the different castes inside the society of the genus *Pheidole* [33, 34]. This beetle is able to use the same anatomical structure (possibly moving the hind legs singly or in combination or in any other way) to “speak” different “languages” by modulating the emissions of various types of pulses. Thus, the beetle can mimic the language of all the ant castes (Fig 3 and Table 2). Difficulties in rearing these insects prevented us from performing laboratory observations to assess if *Paussus* produces different stridulations in different circumstances (e.g. interactions with different ant castes) or if the beetle is able to choose which stridulation to emit as a function of its needs (e.g. for being accepted into the nest; for getting free access to the brood chamber, etc.). Our experiments coupled with the beetle's ability to emit at least two different types of stridulation, suggest an important adaptive role of acoustics in facilitating beetle parasitism.

In particular, the emission of single—pulse sounds (Pc) might elevate the beetle's social status to that of the queen, as suggested in bioassays where queens’ and beetle’s single pulse elicited the same “guarding” response in workers. This might allow the beetle to obtain more effective care and to gain free access to any chamber of the nest. Use of stridulations similar to those of soldiers might allow *Paussus favieri* to confound them and then to avoid their attacks [34,35]. Workers responded to each sound stimulus, both to conspecific castes and to *Paussus favieri*, by showing attraction and “nursing” behaviours [34,35]. All these results suggest that, thanks to the emission of pulse trains (Fig 4), *Paussus favieri* is mistaken for a worker, and hence rescued by worker nest-mates.

Our playback experiments reveal that workers are able to distinguish sounds produced by the three ant castes, as they react with different behaviours or different response frequency, as expected if acoustic signals are used to communicate inside the colony.

## Conclusions

Our results support the existence of a very complex acoustical communication system inside the ant colony. *Paussus* use mimetic stridulations to break this channel. The different stridulations emitted by *Paussus* elicit different responses in the ants. This finding is notable not only because it is the first documented case of ant-beetle interaction via the acoustic channel, but because the acoustical behaviour of *Paussus* is much more refined than that previously found in *Maculinea*. Given the large number of insects that live in ant nests as specialised parasites, we suspect that acoustic mimicry has evolved several times in different phylogenetic lineages. The more we investigate the acoustic world of myrmecophiles, the clearer it is that this pathway represents an overlooked route of host colony infiltration.

## Supporting Information

S1 Dataset Playback recording data and values of acoustic parameters used for statistical analyses. (XLSX)

S1 File Audio file of stridulations emit by *Pheidole pallidula* queen. (WAV)

S2 File Audio file of stridulations emit by *Pheidole pallidula* worker. (WAV)

S3 File Audio file of stridulations emit by *Pheidole pallidula* soldier. (WAV)

S4 File Audio file of stridulations emit by *Paussus favieri*. (WAV)

S1 Table Post hoc tests for pairwise differences in the behavioural responses of *Pheidole pallidula* workers to the respective sources of sound. Tukey HSD tests were used for the equality of means. Values in bold indicate differences at p < 0.05.
(DOCX)

S2 Table Post hoc univariate pairwise comparison (Tukey test HSD) of the three sound parameters. Pa = *P*. *favieri* pulse a; Pb = *P*. *favieri* pulse b; Pc = *P*. *favieri* pulse c; Q = *P*. *pallidula* queens; S = *P*. *pallidula* soldiers; W = *P*. *pallidula* workers. Values in bold indicate differences at p< 0.05.
(DOCX)
