# Supplementary material for: The Pied Piper: A Parasitic Beetle’s Melodies Modulate Ant Behaviours
Source: PLoS One. 2015 Jul 8;10(7):e0130541. doi: 10.1371/journal.pone.0130541 (PMC4496082; doi:10.1371/journal.pone.0130541)
Supplement: S1 Table — Tukey HSD tests were used for the equality of means. Values in bold indicate differences at p < 0.05. (DOCX) [file pone.0130541.s006.docx]

**S1 Table.** *P****ost hoc* tests for pairwise differences in the behavioural responses of *Pheidole pallidula* workers to the respective sources of sound.**

| **Source of sounds** | | **DIFFERENCES IN WORKER RESPONSES TO SOUNDS** | | | | |
| --- | --- | --- | --- | --- | --- | --- |
|  |  | Walking | Antennating | Guarding | Digging | Stay |
| *P. favieri* pulse | *P. favieri* train | 0.40 | 0.55 | 0.30 | 0.20 | 0.05 |
| *P. favieri* pulse | Queens | 0.70 | 0.60 | 0.40 | 0.10 | 0.35 |
| *P. favieri* pulse | Soldiers | 0.05 | 1.00 | **0.90** | 0.10 | 0.15 |
| *P. favieri* pulse | Workers | 0.10 | **1.30** | **0.90** | 0 | 0.05 |
| *P. favieri* pulse | Silence | **3.55** | **3.75** | **0.90** | 0.10 | 0.20 |
| *P. favieri* pulse | White Noise | **2.90** | **3.50** | **0.90** | 0.10 | 0.20 |
| *P. favieri* train | Queens | 0.30 | 0.05 | **0.70** | 0.10 | 0.30 |
| *P. favieri* train | Soldiers | 0.45 | **1.55** | 0.60 | 0.30 | 0.20 |
| *P. favieri* train | Workers | 0.50 | **1.85** | 0.60 | 0.20 | 0.10 |
| *P. favieri* train | Silence | **3.95** | **4.30** | 0.60 | 0.30 | 0.25 |
| *P. favieri* train | White Noise | **3.30** | **4.05** | 0.60 | 0.30 | 0.25 |
| Queens | Soldiers | 0.75 | **1.60** | **1.30** | 0.20 | **0.50** |
| Queens | Workers | 0.80 | **1.90** | **1.30** | 0.10 | **0.40** |
| Queens | Silence | **4.25** | **4.35** | **1.30** | 0.20 | **0.55** |
| Queens | White Noise | **3.60** | **4.10** | **1.30** | 0.20 | **0.55** |
| Soldiers | Workers | 0.05 | 0.30 | 0 | 0.10 | 0.10 |
| Soldiers | Silence | **3.50** | **2.75** | 0 | 0 | 0.05 |
| Soldiers | White Noise | **2.85** | **2.50** | 0 | 0 | 0.05 |
| Workers | Silence | **3.45** | **2.45** | 0 | 0.10 | 0.15 |
| Workers | White Noise | **2.80** | **2.20** | 0 | 0.10 | 0.15 |
| Silence | White Noise | 0.65 | 0.25 | 0 | 0 | 0 |

Tukey HSD tests were used for the equality of means.

Values in bold indicate differences at p < 0.05.
