# Supplementary material for: The Pied Piper: A Parasitic Beetle’s Melodies Modulate Ant Behaviours
Source: PLoS One. 2015 Jul 8;10(7):e0130541. doi: 10.1371/journal.pone.0130541 (PMC4496082; doi:10.1371/journal.pone.0130541)
Supplement: S2 Table — Pa = P. favieri pulse a; Pb = P. favieri pulse b; Pc = P. favieri pulse c; Q = P. pallidula queens; S = P. pallidula soldiers; W = P. pallidula workers. Values in bold indicate differences at p< 0.05. (DOCX) [file pone.0130541.s007.docx]

**S5 Table. Post hoc univariate pairwise comparison (Tukey test HSD) of the three sound parameters.**

| **Sources of sounds** | | **Differences** | | |
| --- | --- | --- | --- | --- |
|  |  | **Pulse length** | **Frequency** | **Intensity** |
| Pa* | Pb* | **0.043** | **1075.998** | **3.178** |
| Pa* | Pc* | **0.014** | 30.951 | **25.632** |
| Pb* | Pc* | **0.057** | **1045.048** | **22.454** |
| Q^&^ | S^&^ | 0.002 | 69.252 | **4.520** |
| Q^&^ | W^&^ | **0.010** | **197.845** | **17.978** |
| S^&^ | W^&^ | 0.008 | **267.097** | **13.458** |
| Pa* | Q^&^ | 0.009 | **640.704** | **26.900** |
| Pa* | S^&^ | 0.007 | **571.453** | **22.380** |
| Pa* | W^&^ | 0.001 | **838.549** | **8.922** |
| Pb* | Q^&^ | **0.052** | **435.294** | **23.722** |
| Pb* | S^&^ | **0.050** | **504.546** | **19.202** |
| Pb* | W^&^ | **0.042** | **237.449** | **5.744** |
| Pc* | Q^&^ | 0.005 | **609.753** | 1.268 |
| Pc* | S^&^ | 0.007 | **540.502** | **3.252** |
| Pc* | W^&^ | **0.015** | **807.598** | **16.710** |

*^*^Paussus favieri*: Pa=pulse a; Pb=pulse b and Pc=pulse c.

*^&^Pheidole pallidula*: Q=queens; W=workers and S=soldiers.

Values in bold indicate differences at p< 0.05.
